# A refutation of reported Levallois technology from Guanyindong Cave in south China

**DOI:** 10.1093/nsr/nwz115

**Published:** 2019-08-13

**Authors:** Feng Li, Yinghua Li, Xing Gao, Steven L Kuhn, Eric Boëda, John W Olsen

**Affiliations:** 1 Key Laboratory of Vertebrate Evolution and Human Origins of the Chinese Academy of Sciences, Institute of Vertebrate Paleontology and Paleoanthropology, Chinese Academy of Sciences, China; 2 CAS Center for Excellence in Life and Paleoenvironment, China; 3 School of History, Wuhan University, China; 4 University of Chinese Academy of Sciences, China; 5 School of Anthropology, University of Arizona, USA; 6 C.N.R.S.-UMR7041, Anthropologie des techniques des espaces et des territoires au Plio-Pléistocène, Maison Archéologie et Ethnologie, René-Ginouvès, France

As one of the most complex forms of lithic technology known for the Paleolithic, research on the Levallois stone-knapping method is critically relevant to our understanding of the cognitive abilities and depth of planning capabilities of archaic hominins, as well as the diffusion of knowledge and social learning in Pleistocene societies. For decades, Levallois technology was thought to be absent from East Asia for much of the Pleistocene, appearing only quite late (ca. 50–40 ka BP) [1,2]. In a recent paper, Hu *et al*. claim that Levallois technology was present at the Guanyindong (GYD) site in Qianxi County, Guizhou, Province, South China by at least 170 ka BP [3]. This report claims that the GYD site is an exceptional case, preserving evidence of Levallois technology earlier than 50 ka BP. If verified, it would constitute the earliest occurrence of Levallois technology in East Asia and would considerably expand the known geographical and temporal range of the Levallois method in East Asia. However, there are critical problems with the conclusions reached by Hu *et al*. While we do not dispute the new series of optically stimulated luminescence dating results, we disagree with the authors’ contention that Levallois technology is present at the GYD site.

**Figure 1.**
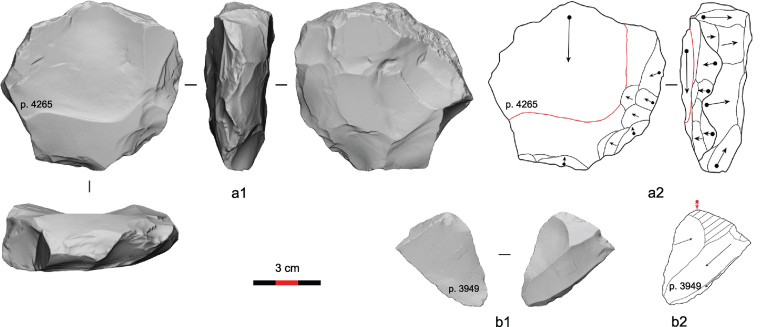
Examples of lithic artifacts from the Guanyindong site, Qianxi County in South China.

The most important problems with the paper by Hu and her colleagues are their misuse of the Levallois concept and the technological misreading of the GYD lithic artifacts. To identify Levallois technology, Hu *et al*. [3] cite the commonly accepted definition of the term. In its broadest sense, the Levallois method is aimed at producing blanks (both flakes and blades) of predetermined shape by careful preparation of the striking platforms and flaking surfaces of cores. The definition that Hu *et al.* base their interpretation on was originally proposed by E. Boëda [4,5]. This definition rests on six specific criteria, including: the presence of two hierarchical, secant surfaces with opposed convexities; the fracture plane of Levallois products being sub-parallel to the intersection of two surfaces; extensive preparation of the striking platform and convexities of the flaking surface; and flaking by means of hard-hammer direct percussion [4,5]. These six criteria were originally proposed to define the technological ‘structure’ of the Levallois method, which would encompass many varieties of the Levallois approach known from the archaeological record of the Lower and Middle Paleolithic. This technological definition was intended to replace conventional reliance on simple morphologies of cores and their products [6], in part because a variety of non-Levallois methods of production can produce a few Levallois-like products in nearly any lithic assemblage.

While Hu *et al*. [3] advocate using the current technological definition of Levallois, it is clear from the text, illustrations and analytical procedures outlined in their paper that they take another approach. Their approach departs not only from the classic typological definition of Levallois [7], but also from a technology-based perspective on the phenomenon [4,5]. The authors adopted what they call a ‘holistic approach’, emphasizing one or two of Boëda’s six technological criteria to identify Levallois technology. The key point is that, whether the six criteria described above are used as a checklist or a guide, archaeologists recognize Levallois as a concept involving a series of technological decisions. These decisions can be understood only by integrating all the information available about a given lithic assemblage [6,8]. One or two criteria are insufficient to define Levallois technology.

For example, the authors stated that cores with hierarchical relationships between their two faces and preferential removals can be considered Levallois [3]. However, many simple cores on flakes yield large, flat preferential flakes but meet none of the other criteria defining Levallois. The authors also treat naturally asymmetrical surfaces as compatible with identification of Levallois technology [3]. The behaviors implied by selecting naturally asymmetrical nodules as blanks for cores are not the same as those implied by intentional shaping of the two faces of Levallois core. In fact, we can find no evidence that this criterion has ever been used to define the Levallois concept. If the authors are determined to use a new technological criterion to refine the current definition of Levallois technology, they should create a new term to label this technology instead of using nomenclature already well established in the literature. Although the method applied by Hu *et al*. [3] is referred to as ‘technological reading’, this approach is in fact highly subjective and arbitrary, no better than an anachronistic typological approach.

At GYD, Hu and her colleagues’ approach has resulted in the misidentification of critical technological characteristics of lithic artifacts. We can cite several instances of misreading lithic artifacts reported in Hu *et al*.’s [3] publication. The most morphologically Levallois-like core in their publication, illustrated as ‘b’ in Fig. 3 (Fig. 1a), exhibits evidence of a large flake detached from one face and smaller scars, presumably indicating core lateral shaping. However, some of the small scars actually overlap the edge of the large flake scar (Fig. 1a), clearly indicating the smaller scars were produced *after* the detachment of the large flake, and so were unrelated to shaping the so-called Levallois surface. One could argue that such edge modifications represent shaping for subsequent removals. However, this trimming was accomplished with alternating removals, which results in two non-hierarchically related core surfaces, contradicting the most essential characteristic of the Levallois concept. More likely, the modified edge of this artifact served as a tool working edge.

The identification of Levallois flakes in Hu *et al*.’s paper [3] can also be questioned. Many of their ‘Levallois products’ exhibit no platform preparation and no shaping of the dorsal face—features typical of Levallois products. For instance, the platform of the so-called Levallois flake illustrated as ‘i’ in Hu *et al.*’s Fig. 3 (Fig. 1b) is plain, without any apparent preparation. The point of percussion is located on the piece’s right corner based upon a clear bulb of percussion visible on the ventral face of the flake, instead of near the middle of what is identified as the flake’s proximal end in the illustration. The flaking direction is clearly oblique to the morphological axis of this flake. When the platform is positioned correctly, the dorsal-scar pattern of this piece does not show systematic organization. The area near the proximal end is flat and is probably formed by a natural fracture plane, and the other two scars display an oblique crossing pattern that can be the result of various non-Levallois knapping techniques. Due to the consistent misreading of the technological characteristics of individual artifacts, the identification of Levallois products in the GYD assemblage reported by Hu *et al*. [3] is not convincing.

One of us (Y.-H.L.) has recently studied the GYD assemblage from a technological perspective. This study showed that production of flakes in the GYD lithic industry was characterized by opportunistic selection of natural technical characteristics on one part of the block to be worked, rather than by the strategic preparation of striking platforms and flaking surfaces. The exploited and unexploited portions of the cores have no apparent association. Different parts of one nucleus may have been flaked if they bore appropriate characteristics, but modifications of various parts of the core are essentially unrelated. Such nuclei exhibiting as ‘additive structure’ are fundamentally distinct from Levallois cores, which reflect an ‘integrated structure’ [9,10]. Occasionally, this approach can produce flakes that superficially resemble Levallois products, but it is a much simpler and less well-organized procedure. A similar situation has been discovered at Zhoukoudian Locality 15, where a few Levallois-like pieces were struck from discoid cores, not from genuine Levallois cores [11].

In conclusion, we believe that, although their work has added valuable new chronometric dates to the corpus of information about the GYD site, there is no evidence of systematic use of Levallois technology in that site’s lithic assemblage, despite Hu and her colleagues’ assertions [3]. It should be noted that our observations are in accordance with those of the original investigators of the GYD site [12] and with those of many other researchers, who conclude that no Levallois or Levallois-like assemblages have yet been reported in southern China [13,14]. Instead, simpler non-Levallois core-flake production dominated the Paleolithic record in the Middle and Late Pleistocene in that region.
